# Transfusion requirements in septic shock (TRISS) trial - comparing the effects and safety of liberal versus restrictive red blood cell transfusion in septic shock patients in the ICU: protocol for a randomised controlled trial

**DOI:** 10.1186/1745-6215-14-150

**Published:** 2013-05-23

**Authors:** Lars B Holst, Nicolai Haase, Jørn Wetterslev, Jan Wernerman, Anders Åneman, Anne B Guttormsen, Pär I Johansson, Sari Karlsson, Gudmundur Klemenzson, Robert Winding, Lars Nebrich, Carsten Albeck, Marianne L Vang, Hans-Henrik Bülow, Jeanie M Elkjær, Jane S Nielsen, Peter Kirkegaard, Helle Nibro, Anne Lindhardt, Ditte Strange, Katrin Thormar, Lone M Poulsen, Pawel Berezowicz, Per M Bådstøløkken, Kristian Strand, Maria Cronhjort, Elsebeth Haunstrup, Omar Rian, Anders Oldner, Asger Bendtsen, Susanne Iversen, Jørn-Åge Langva, Rasmus B Johansen, Niklas Nielsen, Ville Pettilä, Matti Reinikainen, Dorte Keld, Siv Leivdal, Jan-Michael Breider, Inga Tjäder, Nanna Reiter, Ulf Gøttrup, Jonathan White, Jørgen Wiis, Lasse Høgh Andersen, Morten Steensen, Anders Perner

**Affiliations:** 1 Department of Intensive Care, Copenhagen University Hospital, Rigshospitalet, Denmark; 2 Copenhagen Trial Unit, Centre for Clinical Intervention Research, Copenhagen University Hospital, Rigshospitalet, Denmark; 3 Department of Intensive Care, Karolinska University Hospital, Huddinge, Sweden; 4 Department of Intensive Care, Liverpool Hospital, Sydney, Australia; 5 Department of Intensive Care, Haukeland University Hospital and University of Bergen, Bergen, Norway; 6 Section for Transfusion Medicine, Copenhagen University Hospital, Rigshospitalet, Denmark; 7 Department of Intensive Care, Tampere University Hospital, Tampere, Finland; 8 Department of Intensive Care, Landspitalet, Reykjavik, Iceland; 9 Department of Intensive Care, Herning Hospital, Herning, Denmark; 10 Department of Intensive Care, Hvidovre Hospital, Hvidovre, Denmark; 11 Department of Intensive Care, Randers Hospital, Randers, Denmark; 12 Department of Intensive Care, Holbæk Hospital, Holbæk, Denmark; 13 Department of Intensive Care, Kolding Hospital, Kolding, Denmark; 14 Department of Intensive Care, Næstved Hospital, Næstved, Denmark; 15 Department of Intensive Care, Århus University Hospital Nørreborgade, Århus, Denmark; 16 Department of Intensive Care, Bispebjerg Hospital, Bispebjerg, Denmark; 17 Department of Intensive Care, Slagelse Hospital, Slagelse, Denmark; 18 Department of Intensive Care, Vejle Hospital, Vejle, Denmark; 19 Department of Intensive Care, Gentofte Hospital, Gentofte, Denmark; 20 Department of Intensive Care, Stavanger Hospital, Stavanger, Norway; 21 Department of Intensive Care, Södersjukhuset, Stockholm, Sweden; 22 Department of Intensive Care, Horsens Hospital, Horsens, Denmark; 23 Department of Intensive Care, Karolinska University Hospital, Solna, Sweden; 24 Department of Intensive Care, Glostrup Hospital, Glostrup, Denmark; 25 Department of Intensive Care, Slagelse Hospital, Slagelse, Denmark; 26 Department of Intensive Care, Ålesund Hospital, Ålesund, Norway; 27 Department of Intensive Care, Helsingborg Hospital, Helsingborg, Sweden; 28 Department of Intensive Care, Helsinki University Hospital and University of Helsinki, Helsinki, Finland; 29 Department Of Intensive Care, North Karelia Central Hospital, Joensuu, Finland; 30 Department of Intensive Care, Århus University Hospital Skejby, Århus, Denmark; 31 Department of Intensive Care, Sønderborg Hospital, Sønderborg, Denmark; 32 Department of Intensive Care, Centrallasarettet, Växjö, Sweden

**Keywords:** Sepsis, Septic shock, Intensive care medicine, Red blood cell transfusion, Fluid therapy

## Abstract

**Background:**

Transfusion of red blood cells (RBC) is recommended in septic shock and the majority of these patients receive RBC transfusion in the intensive care unit (ICU). However, benefit and harm of RBCs have not been established in this group of high-risk patients.

**Methods/Design:**

The Transfusion Requirements in Septic Shock (TRISS) trial is a multicenter trial with assessor-blinded outcome assessment, randomising 1,000 patients with septic shock in 30 Scandinavian ICUs to receive transfusion with pre-storage leuko-depleted RBC suspended in saline-adenine-glucose and mannitol (SAGM) at haemoglobin level (Hb) of 7 g/dl or 9 g/dl, stratified by the presence of haematological malignancy and centre. The primary outcome measure is 90-day mortality. Secondary outcome measures are organ failure, ischaemic events, severe adverse reactions (SARs: anaphylactic reaction, acute haemolytic reaction and transfusion-related circulatory overload, and acute lung injury) and mortality at 28 days, 6 months and 1 year.

The sample size will enable us to detect a 9% absolute difference in 90-day mortality assuming a 45% event rate with a type 1 error rate of 5% and power of 80%. An interim analysis will be performed after 500 patients, and the Data Monitoring and Safety Committee will recommend the trial be stopped if a group difference in 90-day mortality with *P* ≤0.001 is present at this point.

**Discussion:**

The TRISS trial may bridge the gap between clinical practice and the lack of efficacy and safety data on RBC transfusion in septic shock patients. The effect of restrictive versus liberal RBC transfusion strategy on mortality, organ failure, ischaemic events and SARs will be evaluated.

**Trial registration:**

ClinicalTrials.gov: NCT01485315. Registration date 30 November 2011. First patient was randomised 3 December 2011.

## Background

The first line treatments for patients with septic shock are antibiotics, source control and resuscitation with intravenous fluids and vasopressor/inotropic drugs to optimise circulation and organ perfusion. These interventions may be supplemented with red blood cells (RBCs) in case of anaemia and persistent hypoperfusion [1].

It is known from large prospective studies in European and North American intensive care units (ICUs) that anaemia is very common in critically ill patients; 65% of critically ill patients have haemoglobin (Hb) level <12 g/dl (7.4 mM) at time of admission to the ICU and a mean admission Hb level of 11.3 g/dl (7 mM) [2,3]. As a result of this, 40 to 50% of patients admitted to ICUs are transfused with RBCs during their stay, and 90% of transfusions are administered to non-bleeding patients with a mean of 5 units of RBC per transfused patient. The mean pre-transfusion Hb level - the trigger - in ICU patients is reported to be around 8.5 g/dl (5.3 mM) [4,5].

RBC transfusion has traditionally been perceived as an effective treatment for patients with anaemia - especially for patients with clinical signs of reduced tissue oxygenation [6]. The main function of RBCs is to transport oxygen from the pulmonary to the peripheral capillaries. Thus, RBCs are administered to increase Hb levels and thus blood oxygen-carrying capacity in patients with sepsis to prevent tissue hypoxia and thereby multiple organ failure. However in patients with septic shock, oxygen delivery (DO_2_) may increase after RBC transfusion without a corresponding increase in oxygen consumption (VO_2_) [7]. Several mechanisms may lie behind this observation.

Firstly, tissue hypoxia in the early phase of sepsis might be due to heterogeneous perfusion (stagnant hypoxia) [8], which may not be amenable to RBC transfusion. Secondly, the stored RBCs may not deliver oxygen as efficient as native cells, perhaps due to biochemical and rheological changes of the RBC suspension *ex vivo*, so called storage lesion [9,10]. Thirdly, organ cells may be unable to exploit the increase in oxygen tension due to mitochondrial changes and such cytopathic hypoxia [11] will not be amenable to increased DO_2_ in general [12,13].

In addition, RBC suspensions may have immunomodulatory properties, which can be harmful to patients with sepsis [14,15].

The two trials randomising adult patients with sepsis to different RBC transfusion strategies have shown divergent results. The trial by Rivers and colleagues indicated increased survival with a complex early goal-directed protocol (the goal being central venous oxygen saturation (ScvO_2_) ≥70%) including RBC transfusion. Mortality was 31% with early goal-directed therapy versus 47% in controls, but the role of RBC transfusion was unclear since transfusion was given only if hypoperfusion persisted after initiation of mechanical ventilation, fluid and administration of inotropic drugs [16]. On the other hand, the Transfusion Requirements in Critical Care (TRICC) trial randomising 838 resuscitated and normovolaemic ICU patients to a transfusion trigger of either 7 g/dl (4.3 mM) (restrictive) or 10 g/dl (6.2 mM) (liberal) found no significant difference in the primary outcome measure - 30-day mortality - between the two groups [17]. Hospital mortality was higher in the liberally transfused patients, who also had significantly more cardiopulmonary complications in the ICU than those in the restrictive group. Predefined subgroup analyses indicated lower mortality in the restrictive transfusion group in younger (age <55 years) and less severely ill patients (Acute Physiology and Chronic Health Evaluation (APACHE) II-score <20).

The results of this trial should be interpreted with caution, since the planned inclusion of 1,600 patients was not achieved due to slow recruitment. The patient population may not be representative for ICU patients in general because cardiovascular disease was more common in the excluded patients than in the included. Thus, potential negative effects of restrictive transfusion practice in cardiac patients may not have been discovered. Furthermore the patients were transfused with non-leuko-depleted RBCs stored in citrate suspension, making it difficult to adapt the results to clinical practice today, where pre-storage leuko-depleted blood is widely used. Finally, all patients were resuscitated and deemed normovolaemic by the clinicians when randomised and therefore less likely to have tissue hypoperfusion.

A cochrane review published in 2012 [18] found 19 randomised clinical trials, involving 6264 patients, examining the effects of transfusion thresholds on different outcome variables. Three trials included intensive care patients and one of these was in paediatric patients. Most of the mortality data (52%) came from the TRICC trial [17]. In this review, restrictive transfusion strategy did not increase the rate of adverse events (that is, mortality, cardiac events, myocardial ischaemia, stroke, pneumonia and thromboembolism) compared to liberal transfusion strategies. Furthermore restrictive transfusion strategies were associated with a significant reduction in the rate of infections and hospital mortality but not 30-day mortality. The authors of the Cochrane review concluded that current evidence supports the use of a restrictive transfusions strategy for most patients, including patients with pre-existing cardiovascular disease, but more research is needed to evaluate the effects of restrictive transfusion in high-risk patients.

Taken together, RBC transfusion for patients with septic shock remains controversial because important efficacy and safety questions have not been firmly addressed in previous trials. The optimal Hb-guided transfusion strategy that outbalances risk and benefit remains to be established in this subgroup of high-risk patients.

### Aim

The aim of the TRISS trial is to assess the effects of two well-defined Hb-trigger guided transfusion strategies on mortality and morbidity in ICU patients with septic shock.

## Methods/Design

This is a multicentre trial with computer generated allocation sequence, centralised stratified randomisation, concealed allocation, and blinded outcome assessment of patients with septic shock. The patients will be stratified by centre and by the presence or absence of haematological malignancy and randomised 1:1 to RBC transfusion at Hb ≤7 g/dl (4.3 mM) or Hb ≤9 g/dl (5.6 mM). The latter stratification variable was chosen because these patients have very high mortality rates (70% at 90 days in the 6S trial [19]) and will only be included at few trial sites. Therefore, centre stratification alone may not ensure equal distribution of these patients into the two trial arms.

## Hypothesis

The present evidence is not sufficient to support either restrictive or liberal transfusion strategy in ICU patients with septic shock underlining the need for this trial. The transfusion triggers chosen for this trial are well within the range of the current transfusion practice. We do not have *a priori* expectations on superiority/inferiority of one of the transfusion strategies in this trial. However, a restrictive transfusion strategy in patients with septic shock has the potential to reduce the relative risk of death by 20% (9% absolute risk reduction) compared with a liberal strategy based on the subgroup of patients with severe infection in the TRICC trial [17].

### Trial interventions

Enrolled patients are given a RBC transfusion when they reach their assigned trigger level (Hb ≤9 g/dl (5.6 mM) or 7 g/dl (4.3 mM)) during the entire ICU stay to a maximum of 90 days after randomisation. After ICU discharge or 90 days after randomisation transfusions are given at the discretion of the clinicians despite group allocation. If the patient is readmitted to the ICU within 90 days after randomisation, the Hb-trigger value assigned at randomisation will be reused regardless of the readmission diagnose or status.

RBCs will be transfused as single units followed by renewed Hb assessment by point-of-care testing within 3 hours of termination of the last transfused unit or before the initiation of a new transfusion. All other interventions will be at the discretion of clinicians.

**Figure 1:**
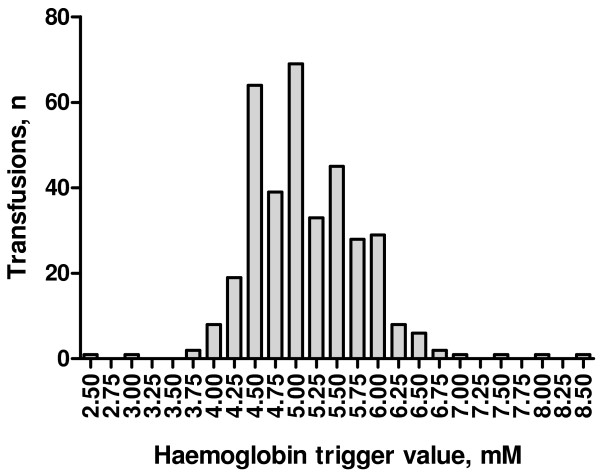
**Transfusion trigger levels in Denmark.** The figure shows the lowest haemoglobin level measured 0 to 2 hours before red blood cell (RBC) transfusion in 213 consecutive septic shock patients in 7 Danish ICUs. The data represent 358 transfused units of saline-adenine-glucose-mannitol (SAGM) [20]. To convert values in mM to g/l multiply with 1.6.

The choice of the two transfusion triggers is based on data from observational studies representing current transfusion practice in septic shock patients in Scandinavia [5,20] [Figure 1].

All trial sites will use pre-storage leuko-depleted RBCs suspended in saline-adenine-glucose-mannitol (SAGM). The intervention is to be administered as an intravenous infusion after making sure that a match of recipient and donor blood has been carried out. The exact amount of blood (ml) in each unit and the exact amount of blood transfused will be recorded by the clinical staff on a transfusion registration sheet when SAGM transfusions are initiated and terminated.

### Concomitant medication/treatment

All other interventions will be at the discretion of the clinicians.

### Inclusion criteria

Adult (age 18 years or above) patients in the ICU who:

•Have anaemia (Hb ≤9 g/dl (5.6 mM))

•AND

•Fulfil the criteria for septic shock [see full criteria in Additional file 1] [21]:

a) Fulfil at least two systemic inflammatory response syndrome (SIRS) criteria within the last 24 hours [22]

And

b) Has a suspected or verified focus of infection

And

c) Has hypotension (systolic or mean arterial blood pressure ≤90 mmHg or ≤70 mmHg, respectively) despite fluid therapy OR requires infusion of vasopressor/inotropic agents to maintain blood pressure.

### Exclusion criteria

Patients fulfilling one or more of the following criteria will not be included:

•Documented wish against transfusion

•Previous SAR with blood products (except transfusion-associated circulatory overload (TACO))

•Presence of ongoing myocardial ischaemia at time of randomisation ((defined as: 1) Patients diagnosed with : a) acute myocardial infarction (ST-elevation myocardial infarction or non-ST elevation myocardial infarction) or b) unstable angina pectoris during current hospital admission, according to the criteria in the clinical setting in question (for example, elevated biomarkers, ischaemic signs on ECG, clinical presence) **AND** 2) the patient has received treatment, initiated during current hospital admission, as a consequence of this (reperfusion strategies (PCI/thrombolysis) or initiated/increased antithrombotic drug treatment))

•Life-threatening bleeding at time of randomisation defined as: (1) Presence of haemorrhagic shock, as judged by research or clinical staff OR (2) the need for surgical procedure, including endoscopy to maintain Hb levels

•RBC transfusion during current ICU admission, administered before randomisation

•Withdrawal from active therapy or brain death

•Acute burn injuries regardless of severity or total burn surface area

**Figure 2:**
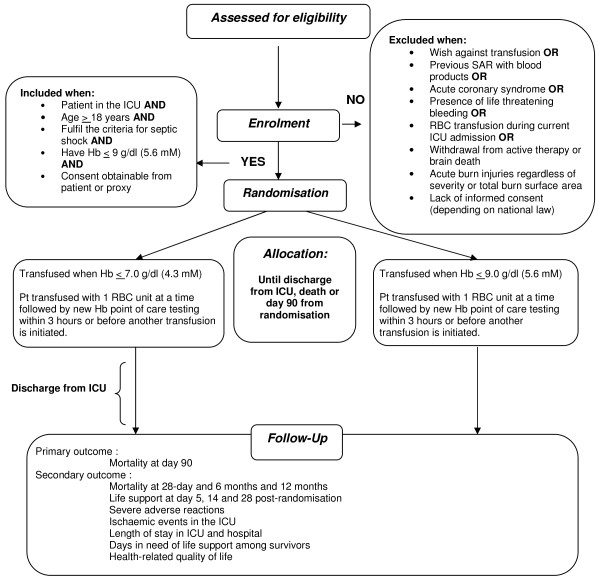
Transfusion requirements in septic shock (TRISS) trial flow diagram.

•Lack of informed consent (in Sweden, Norway, Finland and Iceland consent is obtained from next of kin prior to randomisation; in Denmark delayed consent is obtained from next of kin and general practitioner after randomisation), [Figure 2].

### Randomisation

Screening and randomisation are centralised, web-based, and accessible 24-hour around-the-clock according to the allocation list, the stratification variables and varying block size created by the Copenhagen Trial Unit (CTU) and kept secret from the investigators to allow immediate and concealed allocation to the intervention.

### Primary outcome measure

•Mortality 90 days post-randomisation

### Secondary outcome measures

•Mortality within the whole observation period reported at day 28, 6 months and 1 year after randomisation of the last patient

•Life support at day 5, 14 and 28 post randomisation as use of mechanical ventilation, renal replacement or vasopressor/inotropic therapy [23]

•Severe adverse reactions in the ICU including anaphylactic/allergic reactions, acute haemolysis, transfusion-associated acute lung injury (TRALI), and transfusion associated circulatory overload (TACO)

•Ischaemic events in the ICU including acute myocardial-, cerebral-, intestinal- and acute peripheral limb ischaemia

•Days alive and out of hospital

•Days alive without mechanical ventilation in the 90 days after randomisation

•Days alive without dialysis/haemofiltration in the 90 days after randomisation

•Days alive without vasopressor/inotropic therapy in the 90 days after randomisation

•Health-related quality of life (HRQoL) for Danish patients assessed using the Physical and Mental Component Summary (PCS and MCS) scores of the country specific Short Form health survey questionnaire (SF-36 [24,25]) one-year after randomisation

### Blinding

It will not be feasible to mask the assigned transfusion strategy from health care providers. Consequently, clinical staff caring for the patients will be aware of the allocation and correlated intervention bias as well as other bias mechanisms that may not be controlled. However, information on whether the primary outcome of death occurred will be acquired through the National Civil Registries immediately before the interim analysis and the final data analyses. Thus, steering committee members or investigators will have no knowledge to enable them to compare outcome variables with intervention group allocation for any patient. The independent trial statistician will also be blinded for the allocation during analysis. Information on the secondary outcomes, except long-term mortality, will be provided by the local investigators from patient notes, but the statistician doing the final analyses will be blinded for the allocation. The members of the data and safety monitoring committee (DMSC) will remain blinded unless they request otherwise and after the interim analysis has provided strong indications of one intervention being beneficial or harmful.

### Participant withdrawal

Patients may be withdrawn from the trial at any time if consent is withdrawn by the person(s), who has given proxy consent or by the patient.

The person(s) demanding withdrawal from trial intervention will be asked for permission to continue data registration. In the event the patient does not prohibit obtaining information on the primary outcome measure, it will be obtained centrally. Thus, there may be the following types of withdrawal:

•From intervention only (allowing for all data registration and follow-up)

•From intervention and further registration (but maintaining already registered data and centralised outcome assessment)

•From intervention, further registration, follow-up, and previously registered data demanding deletion of already registered data. Only the patient can demand deletion of already registered data and only if the patient did not consent previously.

If patients deny use of data, we are obliged to delete all data. We expect few of these denials and that the trial will continue until full sample size has been reached to maintain statistical power without further violating the randomisation scheme [19].

Patients who are transferred to another ICU will be withdrawn from the transfusion protocol. However, if the new ICU is an active trial site, the allocated transfusion Hb-trigger level will be maintained in this new ICU. In any case, patients who are transferred to another ICU will be followed up for the primary outcome measure.

### Suspension of protocol

The protocol may temporarily be suspended for the individual patient, at the discretion of the attending doctor, in case of [see Additional file 2 for details]:

•Life-threatening bleeding or

•Ischaemic events

After stabilisation in these instances, the patient will re-enter the protocol. For non-life-threatening bleeding, including surgical procedures, the protocol will be maintained.

### Severe adverse reactions

Serious Adverse Reactions will be registered and are [see Additional file 3 for details]:

•Anaphylactic/allergic reactions

•Severe haemolytic complications

•Transfusion associated acute lung injury (TRALI)

•Transfusion associated circulatory overload (TACO)

Patients who experience a SAR will not be withdrawn from the trial protocol.

### Use of hydroxyethyl starch

The recently completed Scandinavian Starch for Severe Sepsis/Septic Shock (6S) trial showed significantly increased mortality (51% versus 43%, *P* = 0.03) and use of renal replacement therapy (RRT) (22% versus 16%, *P* = 0.04) in patients with severe sepsis or septic shock who received HES 130/0.42 compared with those receiving Ringer’s acetate [18]. These findings are supported by other recent trials [26,27]. Therefore, we prohibit the use of all starch preparations (that is, Voluven™, Tetraspan™ etcetera) in the TRISS trial.

### Statistics

For this study, 2 × 500 patients will be needed to show a 9% absolute risk difference in 90-day mortality (relative risk reduction of 20% with restrictive transfusion among patients with severe infection in the TRICC trial) and mortality of 45% (obtained from 41% in the East Danish Septic Shock Cohort [28] and 51% in a later cohort of septic shock patients in Danish ICUs [29], alpha of 0.05 (two-sided) and power of 80% (1-beta). The Trial Sequential Analysis [30] showed that at least an information gap of 1,000 patients may be expected assuming a 19% relative risk reduction of mortality, and a diversity (D-square) of 0%, and a control event percentage of 11% as found in the traditional meta-analysis of the relevant trials. A type 1 and 2 error rate of 5% and 10%, respectively, were used for the trial sequential analysis [see Additional file 4].

The primary analyses will be by intention-to-treat comparing the two groups by logistic regression analysis for binary outcome measures adjusted for stratification variables (site and presence of haematological disease). An unadjusted Chi-square test for differences in the binary outcomes will be done as a co-primary analysis.

We will perform per protocol analyses of the primary outcome and the most important secondary outcomes excluding patients with one or more major protocol violations [see Additional file 5]. SAS software, version 9.3 (Cary, NC, US) will be used for data management and analysis.

### Interim analysis

An interim analysis will be conducted when patient number 500 has been followed for 90 days [see Additional file 6 for details].

The independent DMSC will recommend pausing or stopping the trial if it finds:

•A group difference in the primary outcome measure *P* <0.001 (Haybittle-Peto criterion) [31,32]. If an analysis of the interim data from 500 patients fulfils the Haybittle-Peto criterion the inclusion of further patients will be paused and an analysis including patients randomised during the analysis period will be performed. If this second analysis also fulfils the Haybittle-Peto criterion or if the group sequential monitoring boundaries are reached the DMSC will recommend stopping the trial.

•Results from other trials combined with the interim analysis from the TRISS trial show clear benefit or harm with RBC transfusion in meta-analysis using trial sequential analysis [30] with a diversity-adjusted required information size [33] based on an *a priori* relative risk reduction of 10%, an overall type 1 error rate of 5% and a type 2 error rate of 20% (power of 80%) and a control event proportion percentage of 45%.

### Intervention accountability

Every patient will be allocated a transfusion registration sheet. This will be kept on site in the site master file. The transfusion registration sheet will include the allocated patient screening number, time for initiation of transfusion and unit volume.

### Registration

Data will be registered into the electronic web-based case report form (eCRF) from patient notes (source data) by trial site personnel. The CTU in cooperation with the coordinating investigator will establish the trial database from an export of data from the eCRF. Paper CRF will be used in case of technical difficulties. Any deviation from the protocol will be captured either in the eCRF or in notes-to-file. Data registration is performed at each participating site by trained personnel.

### The following data will be registered

Pre-randomisation and baseline characteristics: Basic patient characteristics (national identification number or date of birth and site of inclusion (dependent on national law), sex, estimated weight, suspected or confirmed site of infection, surgery during current admission (emergency, elective or not), date of admission to hospital and date and time of admission to ICU and from where the patient was admitted to ICU, co-morbidity (haematological malignancy or not (assessed at screening), chronic obstructive pulmonary disease, asthma or other chronic lung disease or not, cardiovascular disease or not (defined by history of acute myocardial infarction, stable/unstable angina pectoris, previous coronary intervention (CABG or PCI), chronic heart failure (NYHA class 3 to 4) [34], vascular disease (as previous central (aortic or iliac) or peripheral vascular intervention) or ischaemic stroke (including infarction and transitory cerebral ischaemia) and use of RRT.

24 hours prior to randomisation: Lowest/highest Hb level, volume of transfused blood components (specified as RBCs, plasma and platelets), lowest values of ScvO_2_, highest value of p-lactate and data for Simplified Acute Physiology Score (SAPS) 2 [35] and Sepsis-related Organ Failure Assessment (SOFA) scoring [36].

Daily during the entire ICU stay: Hb-levels (daily minimum, maximum and number of assessments), volumes of transfused blood products (RBCs, plasma and platelets), time for initiation of RBC transfusion, unit ID, blood storage time, fluid in-/output, renal replacement therapy or not, vasopressor/inotropic infusion or not, mechanical ventilation or not, lowest PaO_2_/FiO_2_, lowest ScvO_2_, highest p-lactate, surgery or not, any bleeding, ischaemic events, severe adverse reactions (SAR), and decision on not resuscitate in case of cardiac arrest.

90 days after randomisation: Survival status and hospital discharge status obtained from hospital or civil registries, and date of death if the patient has deceased.

Last day of any of the following interventions if the patient was discharged from the trial ICU receiving any of these: Renal replacement therapy, vasopressor/inotropic infusion and mechanical ventilation. We plan to perform a landmark mortality analysis for all randomised patients with a follow-up for each patients of 90 days, the primary analysis will be a logistic regression analysis adjusted for stratification variables. Further, we plan to perform survival analyses including Kaplan-Meier estimates within the total observation time. That is until the last randomised patient has been followed for 3 month. Within the total observation time we will also perform an adjusted proportional hazards analysis (Cox regression analysis), provided the criterion on proportional hazards is fulfilled, adjusting for all the pre-specified covariates listed in the protocol [37-39].

Twelve months after randomisation: Survival status obtained from hospital or civil registries and date of death if the patient is deceased. Days in need of life support (mechanical ventilation, renal replacement or vasopressor/inotropic therapy) in survivors: Status obtained from hospital or civil registries. Health-related quality of life in survivors obtained by posting of the SF-36 questionnaire followed by phone contact if the patient does not reply.

### Data handling and record keeping

Data will be handled according to the data protection agencies of the different countries. All original records (including consent forms, eCRFs, and relevant correspondences) will be archived at trial sites or at CTU for 15 years. The clean electronic trial database file will be anonymised and delivered to the Danish Data Archive and maintained for 15 years.

### Monitoring

Monitoring will adhere to good clinical practice (GCP [40]) principles and be performed according to a predefined monitoring plan including the following issues:

•Initiation visits at all sites

•For all patients: Documented informed consent

•For all patients: Primary outcome according to national or hospital registries

•For 100 patients being the first two patients at each trial site, and another two patients randomly chosen at each trial site: Documented delivery or non-delivery in the eCRF of the intervention according to the protocol compared with source data being patients’ hospital records

•The coordinating centre will continuously monitor that all eCRFs are fulfilled according to the protocol

•Termination visit at all sites: Documenting informed consent for all participants.

A centralised day-to-day monitoring of the eCRF and adherence to the protocol (for example, the ability of individual centres to transfuse at assigned transfusion values only) will be done by the coordinating investigator or his delegates. Additional monitoring visit will be made to selected sites if the steering committee finds this necessary based on monitoring findings.

### Ethical considerations

The trial will be conducted in adherence to the current version of the Helsinki Declaration [41] and to the standards of GCP. Screening of patients will only start after approval by the ethics committee and data protection agency in the countries of the trial sites.

There is no conclusive evidence from RCTs on the potential benefit or risk of RBC transfusion in adults with septic shock. RBC transfusion is part of the current treatment of septic shock, and the Hb-trigger values chosen for the present trial are well within those observed in clinical practice. Thus, the participants will not be exposed to known risks when included into the trial.

Furthermore, the research question is in the public’s interest and the trial design will provide meaningful data with the potential to reach statistical significance and therefore lead to the acceptance or rejection of the null hypothesis.

### Ethical approvals

By 8 January 2013 the study had been approved by: (Denmark) De Videnskabetiske Komiteer - Region Hovedstaden (H-3-2011-114); (Sweden) Regionala etikprövningsnämnden i Stockholm (2011/2:8) (2012/814-32); (Norway) Regionale Komiteer For Medicinsk og Helsefaglig Forskningsetikk (2011/2270/REK vest); (Finland) Tampereen Yliopistollisen Sairaalan Erityisvastuualueen Alueellinen Eettinen Toimikunta (R12269).

### Informed consent

The majority of patients assessed for enrolment in the trial will be unable to give informed consent because of severe illness or as a consequence of the treatment (sedation). Some patients will thus be randomised and enrolled before obtaining informed consent if applicable by national law and after approval by the Ethics Committee for each of the participating ICUs.

There is no alternative to this approach as no clinically relevant model of septic shock exists and no conscious patients have the combination of severe infection, shock and multiple organ failure.

Furthermore, septic shock is an acute life-threatening condition and rapidly initiated resuscitation according to guidelines [1] is important to give the patient the best chance of survival. It would therefore be unacceptable to delay initiation of treatment while awaiting informed consent.

As soon as possible after enrolment proxy consent will be obtained from the patient’s next of kin or general practitioner/regional medical officer of health according to national law. Patients who regain consciousness, will be asked for informed consent as soon as possible.

### Duration

**Figure 3:**
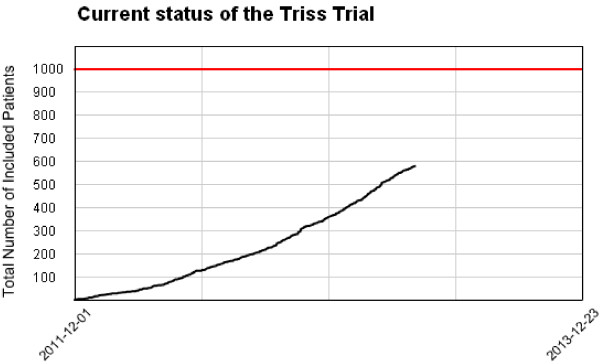
**Trial status.** Print from the ransfusion equirements n eptic hock (TRISS) electronic Case Record Form showing the trial status on 19 April 2013.

Patients from 31 Scandinavian ICUs are expected to be included during a 2-year inclusion period starting December 2011. Based on data from 6S trial it is realistic to include a mean of two patients per ICU per month [19] [Figure 3].

### Low recruitment contingency plan

In case of low recruitment we will involve new trial sites to reach the goal of including 1,000 patients within the 2-year time period.

### Co-enrolment

We will assess the eligibility of patients included in the TRANSFUSE trial (ClinicalTrials.gov identifier: NCT01638416) but not of patients included in the ARISE trial (ClinicalTrials.gov identifier: NCT00975793).

### Timeline

•2011: Protocol, approvals from ethical committees, trial tool development (eCRF and randomisation system)

•2012 to 2013: Inclusion of patients

•Mid-2013: Interim analyses

•2014: The database is expected to be closed in March 90 days after the inclusion of the last patient. Data analyses and writing of the manuscript will be in April followed by submission for publication shortly thereafter

### Trial organisation

**Figure 4:**
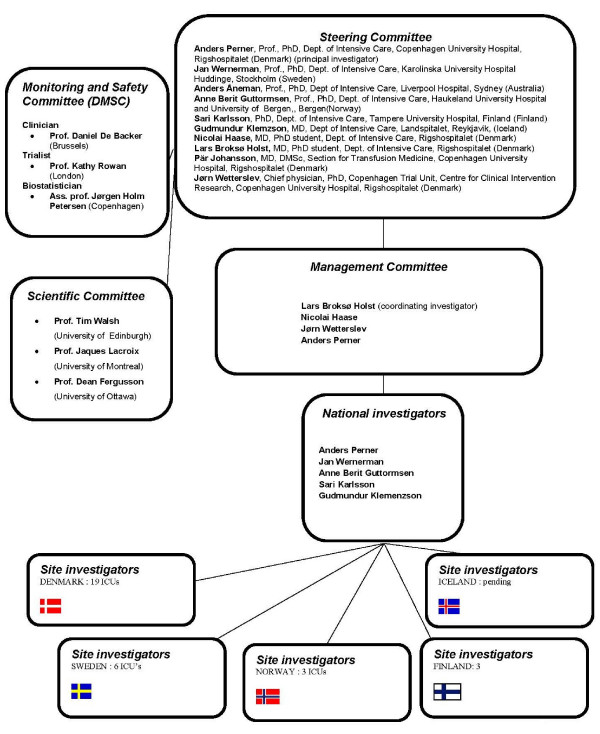
Organisational diagram.

This trial is investigator-initiated as a collaborative research programme between the Scandinavian Critical Care Trials Group, Rigshospitalet, Copenhagen Trial Unit and 31 ICUs in all the Nordic countries. [Figure 4]

### Publication plan

The trial is registered on http://www.clinicaltrials.gov. Upon trial completion the main manuscript will be submitted to one of the major clinical journals regardless of the result, and the results will in any case be published at the SCCTG home page. The Steering Committee will grant authorship in adherence to the Vancouver guidelines [42] and number of patients enrolled by the individual investigator. If a trial site investigator is to gain authorship, the site has to include 25 patients or more. If the site includes 50 patients or more, two authorships will be granted per trial site, 75 patients will give three authorships per trial site and so on.

### Finances

The TRISS trial is funded by the Danish Council for Strategic Research (09–066938) and Copenhagen University Hospital, Rigshospitalet. The funding sources will have no influence on trial design, trial conduct, data handling, data analysis, or publication.

### Perspectives

Severe sepsis affects millions of patients worldwide with high rates of complications and mortality. Outcome differences between therapies for sepsis will therefore have a major impact on global health and healthcare costs. As far as the investigators are aware, no other RCTs are assessing the effects or safety of RBC transfusion in patients with septic shock.

## Discussion

Performing the TRISS trial is in line with recommendations from the 2012 updated Cochrane review [18] and American Association of Blood Bankers [43] guidelines, both stating the need for trials assessing the effects of transfusion triggers in high risk populations.

The TRISS trial may bridge the gap between clinical practice and evidence providing urgently needed data on the efficacy and safety of RBC transfusion for patients with septic shock. The TRISS trial investigators have facilitated a network of Scandinavian ICUs enrolling a high number of patients with septic shock.

### Trial Status

The first patient was randomised 3 December 2011. As of 19 March 2013 31 ICUs are participating, 779 patients have been screened, and 578 patients have been randomised. Ethical approvals in Iceland are pending, and we are expecting 2 to 3 new trial sites to be initiated in the following months.

## Abbreviations

6S: Scandinavian tarch for evere Sepsis/eptic hock trial; ALI: Acute lung injury; ARDS: Acute respiratory distress syndrome; ARR: Absolute risk reduction; CTU: Copenhagen Trial Unit; DMSC: Data monitor and safety committee; DO2: Oxygen delivery; eCRF: electronic (web based) case report form; FiO2: Fraction of inspired oxygen in a gas mixture; Hb: Haemoglobin; ICH-GCP: Guidelines for Good Clinical Practice; ICU: Intensive care unit; PaO2: Partial pressure of oxygen in arterial blood; POC: Point of care; Pt: Patient; RBC: Red blood cells; RCT: Randomised clinical trial; RRR: Relative risk reduction; RRT: Renal replacement therapy; SAE: Serious adverse event; SAGM: Saline-adenine-glucose-mannitol; SAR: Suspected adverse reaction; SC: Steering committee; SCCTG: Scandinavian Critical Care Trials Group; ScvO2: central venous oxygen saturation; SIRS: Systemic inflammatory response syndrome; TACO: Transfusion associated circulatory overload; TRALI: Transfusion associated acute lung injury; TRICC: Transfusion Requirements in Critical Care; TRISS: Transfusion Requirements in Septic Shock trial; Tx: Transfusion; VO2: Oxygen consumption.

## Competing interests

The ICU at Copenhagen University Hospital receives research funds from Fresenius Kabi. Anders Perner, Nicolai Haase, Jørn Wetterslev, Anders Åneman, Anne Berit Guttormsen, and Gudmundur Klemenzson were members of the steering committee of the 6S trial, which was supported by B Braun Medical. The remaining authors declare that they have no competing interests.

## Authors’ contributions

All authors made substantive contributions to the TRISS trial as trial site investigators and revised and gave final approval of the manuscript. LBH drafted the manuscript together with NH, JW and AP. AP is the principal investigator and sponsor of TRISS. LBH designed the trial together with NH, JW, JWM, AÅ, ABG, SC, GK and AP. LBH is coordinating investigator and member of the steering and management committee. AP, JWM, ABG, SC and GK are national investigators.

## Supplementary Material

Additional file 1 Trial criteria for septic shock. Click here for file

Additional file 2 Protocol suspension criteria. Click here for file

Additional file 3 Severe adverse reactions (SARs). Click here for file

Additional file 4 Trial sequential analysis. Click here for file

Additional file 5 Statistical analysis plan. Click here for file

Additional file 6 Charter for the independent Data Monitoring and Safety Committee (DMSC) of the TRISS trial. Click here for file
