# Supplementary material for: Transfusion requirements in septic shock (TRISS) trial - comparing the effects and safety of liberal versus restrictive red blood cell transfusion in septic shock patients in the ICU: protocol for a randomised controlled trial
Source: Trials. 2013 May 23;14:150. doi: 10.1186/1745-6215-14-150 (PMC3679866; doi:10.1186/1745-6215-14-150)
Supplement: Additional file 1 — Trial criteria for septic shock. [file 1745-6215-14-150-S1.pdf]

Additional file 1

Trial criteria for septic shock [21]

(1) ***AT LEAST TWO SYSTEMIC INFLAMMATORY RESPONSE SYNDROME (SIRS) CRITERIA****:*

1. ***CORE TEMPERATURE >38℃ or <36℃*** *. (Core temperature is rectal, urinary bladder, central line, or tympanic). If oral, inguinal or axillary temperatures are used, add 0.5℃ to the measured value. Hypothermia <36℃ must be confi rmed by core temperature. Use the most deranged value recorded* ***in the 24 hours before randomisation****.*
2. ***HEART RATE >90 beats/minute****. If patient has an atrial arrhythmia, record the ventricular rate. If patients have a known medical condition or are receiving treatment that would prevent tachycardia (for example, heart block or beta blockers), they must meet two of the remaining three SIRS criteria. Use the most deranged value recorded* ***in the 24 hours before randomisation****.*
3. ***MECHANICAL VENTILATION*** *for an acute process or respiratory rate > 20 breaths per minute or a* $PaCO_2$ *< 4.3 kPa (32 mmHg). Use the most deranged respiratory rate or* $PaCO_2$ *recorded* ***in the 24 hours before randomisation****.*
4. ***WHITE BLOOD CELL COUNT of >12 x*** 10^9***/l or < 4 x*** 10^9***/l****. Use the most deranged value recorded* ***in the 24 hours before randomisation****.*

AND

(2) ***SUSPECTED OR VERIFIED FOCUS OF INFECTION*** *as either:*

(i) An organism grown in blood or sterile site

OR

(ii) An abscess or infected tissue (e.g. pneumonia, peritonitis, urinary tract, vascular line infection, soft tissue, etc).

AND

(3) ***HYPOTENSION*** *(Systolic blood pressure < 90 mmHg or MAP < 70 mmHg) despite fluid therapy OR* ***VASOPRESSOR/INTROPE*** *infusion to maintain blood pressure.*
