# Supplementary material for: Transfusion requirements in septic shock (TRISS) trial - comparing the effects and safety of liberal versus restrictive red blood cell transfusion in septic shock patients in the ICU: protocol for a randomised controlled trial
Source: Trials. 2013 May 23;14:150. doi: 10.1186/1745-6215-14-150 (PMC3679866; doi:10.1186/1745-6215-14-150)
Supplement: Additional file 2 — Protocol suspension criteria. [file 1745-6215-14-150-S2.pdf]

Additional file 2

Protocol suspension criteria

The protocol may temporarily be suspended for the individual patient, at the discretion of the attending doctor, if the patient is to be transfused with RBC during any of the following events:

- Presence of life-threatening bleeding :

  Defined as the presence of haemorrhagic shock, as judged by research or clinical staff.

- Ischaemic events defined as:

  1. Acute myocardial ischaemia:
     Defined as ***acute myocardial infarction*** *(ST-elevation myocardial infarction and non-ST elevation myocardial infarction) or* ***unstable angina pectoris*** diagnosed during current hospital admission, according to the criteria in the clinical setting in question (e.g. elevated biomarkers, ischaemic signs on ECG, clinical presence) **AND** the patient has received treatment as a consequence of this (reperfusion strategies (PCI/thromobolysis) or initiation/increased antithrombotic drug treatment), during current hospital admission.
  2. Cerebral ischaemia: Verified by CT- or MR scan
  3. Intestinal ischaemia: Verified by endoscopy or open surgery.
  4. Acute peripheral limb ischaemia: Clinical signs AND need of open/percutaneous vascular intervention, amputation or initiation/increased antithrombotic treatment.

The protocol will be resumed promptly once the patient no longer fulfils the suspension criterion. Suspension will not be considered a breach of protocol, and collection of data will continue during the suspension. These patients will be analysed according to their originally assigned groups on an intention-to-treat basis.
