# Supplementary material for: Transfusion requirements in septic shock (TRISS) trial - comparing the effects and safety of liberal versus restrictive red blood cell transfusion in septic shock patients in the ICU: protocol for a randomised controlled trial
Source: Trials. 2013 May 23;14:150. doi: 10.1186/1745-6215-14-150 (PMC3679866; doi:10.1186/1745-6215-14-150)
Supplement: Additional file 3 — Severe adverse reactions (SARs). [file 1745-6215-14-150-S3.pdf]

Additional file 3

Severe adverse reactions (SARs)

Patients will not be withdrawn from the trial protocol if Serious Adverse Reactions (SARs) occur but these will register as:

1. *Anaphylactic/allergic reactions after transfusion (occurrence within 6 hours of transfusion) of RBC*

2. *Severe haemolytic complications after transfusion (occurrence within 24 hours of transfusion) of RBC*

3. *Transfusion-associated acute lung injury (TRALI) after RBC transfusion*

4. *Transfusion associated circulatory overload (TACO) after RBC transfusion*

The occurrence of SARs will be recorded in the eCRF during the ICU stay and compared for the two trial groups by the DMSC at the interim analysis. During the trial, sponsor will send yearly reports on the occurrence of SARs to the DMSC and the ethic committees.

SAEs will not be recorded as an entity, because the majority of septic ICU patients will experience several SAEs during their ICU stay. The most important SAEs will be captured as secondary outcome measures (life support).
