# Supplementary material for: Transfusion requirements in septic shock (TRISS) trial - comparing the effects and safety of liberal versus restrictive red blood cell transfusion in septic shock patients in the ICU: protocol for a randomised controlled trial
Source: Trials. 2013 May 23;14:150. doi: 10.1186/1745-6215-14-150 (PMC3679866; doi:10.1186/1745-6215-14-150)
Supplement: Additional file 4 — Trial sequential analysis. [file 1745-6215-14-150-S4.pdf]

Additional file 4

Trial sequential analysis (TSA)

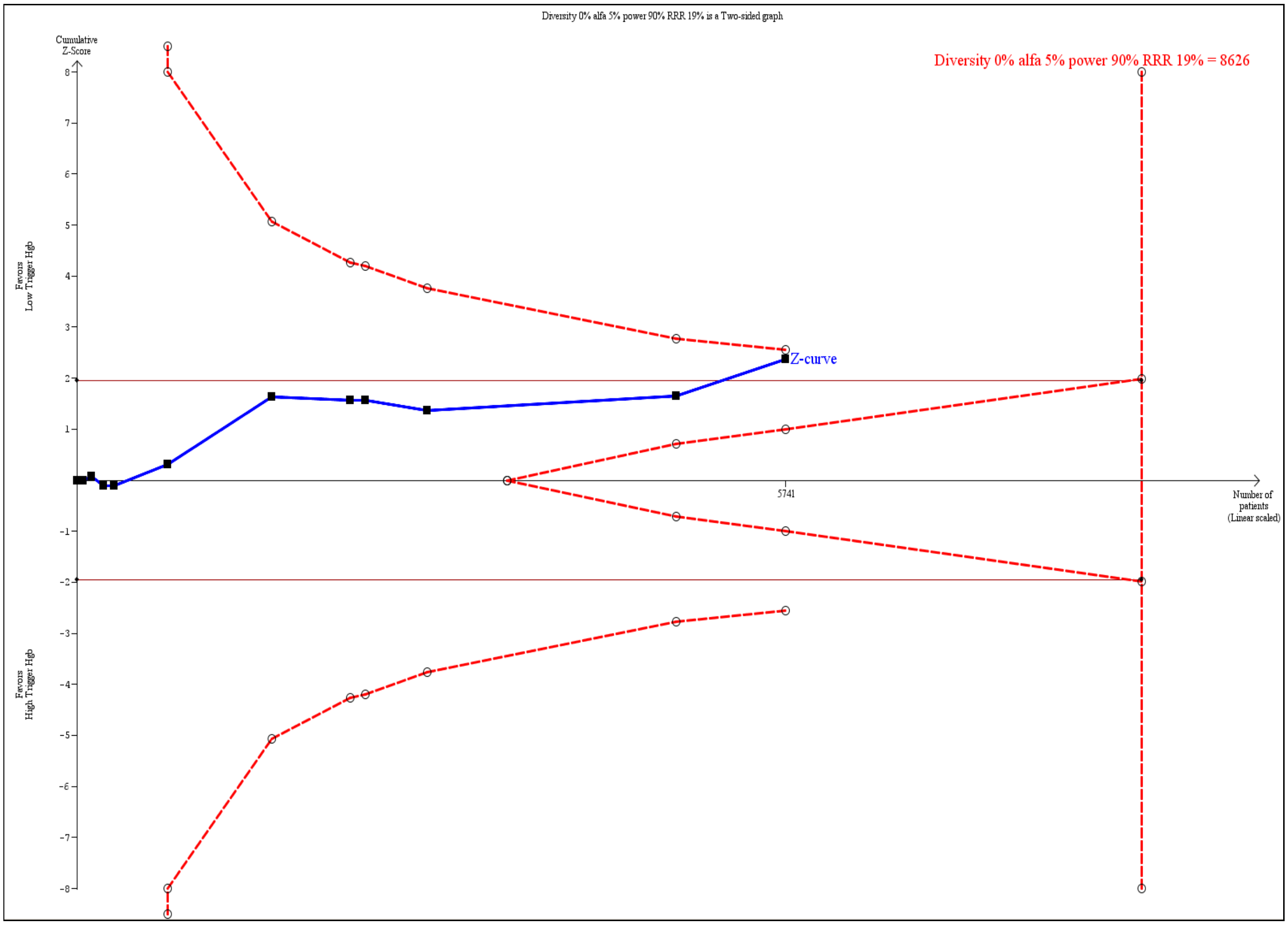

The figure depicts Trial sequential analysis (TSA) of trials comparing the effect of low Hb trigger level with high Hb trigger level on all cause mortality, using a diversity of 0%, an anticipated intervention effect of 19% relative risk reduction, a control event proportion of 11% all of which was indicated in the meta-analysis by Carson et al. 2012 [18] up-dated with Villanueva et al. NEJM 2013 [44]. A type 1 and 2 error risk of 5% and 10% respectively was used. The required information size is estimated to be 8236 and neither boundaries for benefit or futility are crossed by the cumulative z-curve suggesting premature declaration of statistical significant benefit in the traditional random-effects meta-analysis (RR=0.81 95% CL 0.67-0.96) TSA adjusted 95% CL being 0.64-1.02.
