# Supplementary material for: Transfusion requirements in septic shock (TRISS) trial - comparing the effects and safety of liberal versus restrictive red blood cell transfusion in septic shock patients in the ICU: protocol for a randomised controlled trial
Source: Trials. 2013 May 23;14:150. doi: 10.1186/1745-6215-14-150 (PMC3679866; doi:10.1186/1745-6215-14-150)
Supplement: Additional file 5 — Statistical analysis plan. [file 1745-6215-14-150-S5.pdf]

Additional file 5

TRISS-Trial Statistical Analysis Plan

<u>Populations</u>

Intention-to-treat population: All randomised patients except those who withdrew their consent for the use of data.

Per-Protocol populations:

Per-protocol #1:

All randomised patients except patients having one or more major protocol violations defined as:

1. One or more RBC transfusions given despite Hb $\geq$ 7.0 g/dl in patients assigned to the restrictive strategy-group

   OR
2. One or more RBC transfusion not given within 24 hours after Hb $\leq$ 9.0 g/dl in patients assigned the liberal strategy-group

   OR
3. Monitoring revealed that one or more in- or exclusion criteria were violated

   OR
4. One or more transfusions of RBC unit(s) destined for another patient

   OR
5. One or more transfusions given despite lack of cross-match between donor and recipient. Administration of 0-neg blood without X-match between donor and recipient will not be regarded as a protocol violation

   OR
6. Any protocol suspension defined as transfusions administered when Hb > trigger level on days with the presence of ischaemic events and/or bleeding

   OR
7. Stopped/withdrawn patients

Per-protocol #2:

All randomised patients except patients having one or more protocol violations defined as

1. One or more transfusions *given* despite the patients Hb level being *above the trigger* level the patient was randomly assigned to

OR

2. *O*ne or more transfusions *not given* within a period of 24 hours after the patient is diagnosed with a Hb level *below the trigger* level that the patient was randomly assigned to

Per-protocol #3:

1. Patients who had one or more bleeding episodes

   OR

2. Patients who had one or more ischaemic episodes

Subgroups:

1. Patients with SAPS II > 53 at baseline
2. Patients age > 70 years
3. Patients with cardiovascular disease

Analyses

Primary analysis:
Will be a logistic regression analysis for binary outcome measures adjusted for stratification variables (site and presence of haematological disease). We will provide an unadjusted Chi-square test for differences in the binary outcomes as well. For rate data the generalized linear model (SAS proc genmod) will be used with distribution Poisson, link=log and offset.

Secondary analysis:
Multiple (logistic) regression and analysis of rate data with the following covariates:

<u>Binary covariates</u>

- Site (stratification variable)
- Hematological malignancy at time of randomisation (stratification variable) Y/N
- Previous cardiovascular disease Y/N
- Surgery during current hospital admission, but prior to randomisation Y/N

<u>Continuous covariate</u>

- Age
- SAPS II in the 24-hours prior to randomisation
- SOFA-score in the 24-hours prior to randomisation
- Hb-level at baseline
- Volume of transfused blood in the 24-hours prior to randomisation

Difference between groups in all-cause mortality within the whole observation period six-month and 1 year after randomisation of the last patient will be analysed using Cox proportional hazards method (Cox regression analysis) using unadjusted analysis and analysis adjusting for the design and patient variables listed above.

Outcomes

Primary outcome measure:

The primary outcome measure of 90-day mortality as retrieved from the National Civil Registries.

Secondary outcome measures:

- Mortality within the whole observation period reported at day 28, six-month and 1 year after randomisation of the last patient.
- Life support at day 5, 14 and 28 (i.e. need of mechanical ventilation, renal replacement therapy or vasopressor/inotropic therapy) post randomisation
- Severe adverse reactions (SARs) in the ICU
- Ischaemic events in the ICU (including myocardial, cerebral, intestinal and peripheral)
- Length of stay in ICU and hospital
- Days in need of life support among survivors
- Health-related quality of life (HRQoL) assessed using the SF-36

Level of statistical significance for all analyses: P = 0.05

Missing Data

Initially, we will perform a complete case-analysis. Then supplementary analyses using imputed data as described below will be performed:

Missing baseline data:

SAPS II
The score is based on values measured in the first 24 hours of ICU admission but we register SAPS II as a baseline score including values from the 24 hours prior to randomisation so patients randomised immediately after ICU admission may have missing values.

SOFA-score
This score does not depend on when the patient is admitted to the ICU but we register SOFA at baseline including values from the 24 hours prior to randomisation. Thus patients randomised immediately after ICU admission may also have missing values in this score.

For SAPS II and SOFA scores day 1 values may reflect patient's condition. However day 1 have variable length as it starts at time of randomisation and ends at the beginning of the next fluid-day. Thus variables may be missing at both baseline and day 1. In these situations data from day 2 may be representative of the patient's condition.

If the frequency of missing data after the above implemented logical imputation is > 5% we will perform *"best"/"worst" case scenarios* where 1) missing SAPS- or SOFA-components in group A will be given to the worst possible score AND missing SAPS- or SOFA-components in group B will be given the best score (zero) or 2) missing SAPS- or SOFA-components in group A will be given the best score (zero) AND missing SAPS- or SOFA-components in group B will be given the worst possible score. If there is no reasonable difference between the results of these two analyses, we will not do further imputation.

If the frequency of missing data after the above implemented "worst-best" scenarios is still > 5% and the complete case analysis is significant at the 10% value or less, we will perform an additional analysis using the multiple imputation method.

If the frequency of missing data after the above implemented "imputations" is > 5%, we will perform an additional analysis using the multiple imputation method.

Missing primary outcome data:
We do not expect missing data on the primary outcome as these will be obtained from hospital or civil registries. Only complete case analysis will be made.

Missing secondary outcome data
Only complete case analysis will be made.

To put significant results into perspective the following sensitivity analysis will be conducted: We define a worst case scenario as one where patients with missing data do not react on the treatment (whatever it may be). Missing data will be imputed according to this scenario. Let P be the estimate of the parameter reflecting the effect of the intervention calculated from the complete case analysis and P-imp be the corresponding estimate calculated from the analysis of the imputed data.
[(P-imp – P)/P-imp]*100% then a ball park figure of the bias is to be expected were the worst case scenario true.
P-imp/ (standard error of P-imp) is calculated and the corresponding p value found to assess the potential impact of this bias on the significance level.
