# Supplementary material for: Transfusion requirements in septic shock (TRISS) trial - comparing the effects and safety of liberal versus restrictive red blood cell transfusion in septic shock patients in the ICU: protocol for a randomised controlled trial
Source: Trials. 2013 May 23;14:150. doi: 10.1186/1745-6215-14-150 (PMC3679866; doi:10.1186/1745-6215-14-150)
Supplement: Additional file 6 — Charter for the independent Data Monitoring and Safety Committee (DMSC) of the TRISS trial. [file 1745-6215-14-150-S6.pdf]

Additional file 6

Charter for the independent Data Monitoring and Safety Committee (DMSC) of the TRISS-trial

Introduction

The Charter will define the primary responsibilities of the DMSC, its relationship with other trial components, its membership, and the purpose and timing of its meetings. The Charter will also provide the procedures for ensuring confidentiality and proper communication, the statistical monitoring guidelines to be implemented by the DMSC, and an outline of the content of the Open and Closed Reports that will be provided to the DMSC.

Primary responsibilities of the DMSC

The DMSC will be responsible for safeguarding the interests of trial participants, assessing the safety and efficacy of the interventions during the trial, and for monitoring the overall conduct of the clinical trial. The DMSC will provide recommendations about stopping or continuing the trial to the Steering Committee (SC) of the TRISS trial. To contribute to enhancing the integrity of the trial, the DMSC may also formulate recommendations relating to the selection/recruitment/retention of participants, their management, improving adherence to protocol-specified regimens and retention of participants, and the procedures for data management and quality control.

The DMSC will be advisory to the SC. The SC will be responsible for promptly reviewing the DMSC recommendations, to decide whether to continue or terminate the trial, and to determine whether amendments to the protocol or changes in trial conduct are required.

The DMSC is planned by protocol to meet physically in order to evaluate the planned interim analysis of the TRISS-trial. The interim analysis will be performed by an independent statistician selected by the members of the DMSC. The DMSC may additionally meet whenever they decide, contact each other by telephone or e-mail in order to discuss the safety for trial participants. Sponsor has the responsibility to report yearly to the DMSC the overall number of Serious Adverse Reactions (SAR).The DMSC can at any time during the trial request the distribution of events, including outcome measures and SARs according to intervention groups. The recommendations of the DMSC regarding stopping, continuing or changing the design of the trial should be communicated without delay to the SC of the TRISS-trial. The SC has the responsibility to inform as fast as possible, and no later than 48 hrs, all investigators of the trial and the sites including patients in the trial the recommendation of the DMSC and the SC decision hereof.

Members of the DMSC

The DMSC is an independent multidisciplinary group consisting of a clinician, a trialist and a biostatistician that, collectively, has experience in the management of ICU patients and in the conduct, monitoring and analysis of randomised clinical trials.

DMSC Clinician

Daniel De Backer (Brussels)

DMSC Trialist

Kathy Rowan (London)

DMSC Biostatistician
Jørgen Holm Petersen, Dept. of Biostatistics, University of Copenhagen

Conflicts of interest
DMSC membership has been restricted to individuals free of conflicts of interest. The source of these conflicts may be financial, scientific, or regulatory in nature. Thus, neither trial investigators nor individuals employed by the sponsor, nor individuals who might have regulatory responsibilities for the trial products, are members of the DMSC. The DMSC members do not own stock in the companies having products being evaluated by the TRISS-trial.
The DMSC members will disclose to fellow members any consulting agreements or financial interests they have with the sponsor of the trial, with the contract research organisation (CRO) for the trial (if any), or with other sponsors having products that are being evaluated or having products that are competitive with those being evaluated in the trial.
The DMSC will be responsible for deciding whether these consulting agreements or financial interests materially impact their objectivity.
The DMSC members will be responsible for advising fellow members of any changes in these consulting agreements and financial interests that occur during the course of the trial. Any DMSC members who develop significant conflicts of interest during the course of the trial should resign from the DMSC.
DMSC membership is to be for the duration of the clinical trial. If any members leave the DMSC during the course of the trial, the SC will appoint the replacement(s).

Formal interim analysis meeting
One 'Formal Interim Analysis' meeting will be held to review data relating to treatment efficacy, patient safety, and quality of trial conduct. The three members of the DMSC will meet when 90-day follow-up data of 500 patients have been obtained.

Proper communication
To enhance the integrity and credibility of the trial, procedures will be implemented to ensure the DMSC has sole access to evolving information from the clinical trial regarding comparative results of efficacy and safety data, aggregated by treatment group (0.1). An exception will be made to permit access to an independent statistician who will be responsible for serving as a liaison between the database and the DMSC.
At the same time, procedures will be implemented to ensure that proper communication is achieved between the DMSC and the trial investigators. To provide a forum for exchange of information among various parties who share responsibility for the successful conduct of the trial, a format for Open Sessions and Closed Sessions will be implemented. The intent of this format is to enable the DMSC to preserve confidentiality of the comparative efficacy results while at the same time providing opportunities for interaction between the DMSC and others who have valuable insights into trial-related issues.

Closed Sessions
Sessions involving only DMSC membership who generates the Closed Reports (called Closed Sessions) will be held to allow discussion of confidential data from the clinical trial, including information about the relative efficacy and safety of interventions. In order to ensure that the DMSC will be fully informed in its primary mission of safeguarding the interest of participating patients, the DMSC will be blinded in its assessment of safety and efficacy data. However, the DMSC can request unblinding from the SC.

Open Reports
For each DMSC meeting, Open Reports will be provided available to all who attend the DMSC meeting. The Reports will include data on recruitment and baseline characteristics, and pooled data on eligibility violations, completeness of follow-up, and compliance. The primary trial statistician will prepare these Open Reports.

Closed Reports will include analysis of the primary efficacy outcome measure. In addition, analyses of the secondary outcome measures and serious adverse events will also be reported. These Closed Reports will be prepared by an independent biostatistician, with assistance from the trial biostatisticians, in a manner that allow them to remain blinded.

The Closed Reports should provide information that is accurate, with follow-up on mortality that is complete to within two months of the date of the DMSC meeting.

The Reports should be provided to DMSC members approximately three days prior to the date of the meeting.

Minutes of the DMSC Meetings
The DMSC will prepare minutes of their meetings. The Closed Minutes will describe the proceedings from all sessions of the DMSC meeting, including the listing of recommendations by the Committee. Because it is likely that these minutes may contain unblinded information, it is important that they are not made available to anyone outside the DMSC.

Recommendations to the Steering Committee
After the interim analysis meeting, the DMSC will make a recommendation to the SC to continue, hold or terminate the trial.

If an analysis of the interim data from 500 patients fulfils the Haybittle-Peto criterion the inclusion of further patients will be paused and an analysis including patients randomised during the analysis and period of pausing the trial will be performed. If this second analysis also fulfils the Haybittle-Peto criterion or the group sequential monitoring boundaries the DMSC will recommend stopping the trial.

If the recommendation is to stop the trial the DSMC will discuss and recommend on whether the final decision to stop the trial will be made after the analysis of all patients included at the time (including patients randomised after patient number 500) and whether a moratorium shall take place (setting the trial at hold) in the further inclusion of patients during these extra analyses. If further analyses of the patients included after 500 patients is recommended rules for finally recommending stopping of the trial should obey the Lan DeMets stopping boundary.

This recommendation will be based primarily on safety and efficacy considerations and will be guided by statistical monitoring guidelines defined in this Charter and the trial protocol.

The SC is jointly responsible with the DMSC for safeguarding the interests of participating patients and for the conduct of the trial. Recommendations to amend the protocol or conduct of the trial made by the DMSC will be considered and accepted or rejected by the SC. The SC will be responsible for deciding whether to continue, hold or stop the trial based on the DMSC recommendations.

The DMSC will be notified of all changes to the trial protocol or conduct. The DMSC concurrence will be sought on all substantive recommendations or changes to the protocol or trial conduct prior to their implementation.

Statistical monitoring guidelines
The outcome parameters are defined in the TRISS-trial protocol. For the two intervention groups, the DMSC will evaluate data on:
<u>The primary outcome measure</u>
Mortality at 90 days after randomisation.

<u>The secondary outcome measures</u>
Need for life-support at days 5, 14 and 28
The occurrence of SARs in ICU
The occurrence of ischaemic events in ICU

The DMSC will be provided with these data from the Coordinating Centre as:
Number of patients randomised
Number of patients randomised per intervention group (0.1)
Number of patients stratified pr. stratification variable per intervention group (0.1)
Number of events, according to the outcomes, in the two groups

Based on evaluations of these outcomes, the DMSC will decide if they want further data from the Coordinating Centre and when next to perform analyses of the data.

For analyses, the data will be provided in one file as described below.

Based on the analyses of the primary outcome measure and SARs, the DMSC will use $P<0.001$ (Haybittle-Peto) as the statistical limit to guide its recommendations regarding early termination of the trial.

Based on 90-day mortality analyses, the DMSC will use $P<0.001$ (Haybittle-Peto) and group sequential monitoring boundaries as the statistical limit to guide its recommendations regarding early termination of the trial.

DMSC should also be informed about all SARs occurring in the two groups of the trial.

The DMSC may also be asked to ensure that procedures are properly implemented to adjust trial sample size or duration of follow-up to restore power, if protocol specified event rates are inaccurate. If so, the algorithm for doing this should be clearly specified.

Conditions for transfer of data from the Coordinating Centre to the DMSC
The DMSC shall be provided with the data described below in one file.

The DMSC will be provided with an Excel database containing the data defined as follows:

Row 1 contains the names of the variables (to be defined below).

Row 2 to N (where N-1 is the number of patients who have entered the trial) each contains the data of one patient.

Column 1 to p (where p is the number of variables to be defined below) each contains in row 1 the name of a variable and in the next N rows the values of this variable.

The values of the following variables should be included in the database:

1: PtID: a number that uniquely identifies the patient.

2: Rdcode: The randomisation code (group 0 or 1) – the DMSC is not to be informed on what intervention the groups received.

3: 90MInd: 90 day-mortality indicator (2 if patient is censored, 1 if patient was dead, and 0 if the patient was alive at day 90).

4: OF5ind: Life support at day 5. (1 if patient in need of life support and 0 if the patient did not).

5: OF14ind: Life support at day 14 (1 if patient in need of life support and 0 if the patient did not).

6: OF28ind: Life support at day 28 (1 if patient in need of life support and 0 if the patient did not).

7: SARInd: Severe Adverse Reaction indicator (1 if patient has had SAR in ICU and 0 if the patient did not).

8: ICHInd: Ischaemic event in ICU (1 if patient has had an ischaemic event in ICU and 0 if the patient did not).
